# Dataset of leisure time among students at Kermanshah University of Medical Sciences and its relationship with health-related quality of life (HRQOL)

**DOI:** 10.1016/j.dib.2018.09.076

**Published:** 2018-09-29

**Authors:** Parvin Abbasi, Neda Kianipour, Gülcan Demir Özdenk, Arash Ziapour

**Affiliations:** a Department of Nursing, School of Nursing and Midwifery, Kermanshah University of Medical Sciences, Kermanshah, Iran; b Students Research Committee, Kermanshah University of Medical Sciences, Kermanshah, Iran; c Vocational School of Health Services in Ahi Evran University, Kırşehir, Turkey; d Kermanshah University of Medical Sciences, Kermanshah, Iran

**Keywords:** Health-related quality of life (HRQOL), Leisure time, University students

## Abstract

The present data article aimed to investigate the leisure time among students at Kermanshah University of Medical Sciences and its relationship with health-related quality of life. In this descriptive and correlational data article, the statistical population consisted of 420 students at faculties of Health, Paramedics, Nursing and Midwifery in Kermanshah University of Medical Sciences who were selected through multi-stage cluster sampling. For data collection, the demographic questions, 36-item short form survey (SF-36) and a researcher-made leisure time questionnaire were utilized. for data analysis, the descriptive and inferential statistics (Pearson correlation coefficient) were employed in the SPSS0.23 Statistics Software. The obtained data of the present data article demonstrated that the means and SDs of students’ leisure time and health-related quality of life measured 3.25 ± 0.61 and 2.50 ± 0.41, respectively. Also, the obtained data indicated that the quality of life significantly and positively correlated with students’ leisure time. In addition, the obtained data showed that in-person communication had the highest relationship with the total quality of life. In contrast, no relationship was found between the artistic activities and the total quality of life.

**Specifications table**

**Table:** 

Subject Area	Social Sciences
Subject Area	Health Promotion
Type of Data	Tables
How data was acquired	For data collection, 45-item Leisure Time Questionnaire and the 36-item Short Form Survey (SF-36)were used. The statistical population consisted of 420 students at faculties of Health, Paramedics, Nursing and Midwifery in Kermanshah University of Medical Sciences in 2017.
Data Format	Raw Data and Analyzed data
Experimental Factors	The reliability and validity of the questionnaire were evaluated using Cronbach׳s alpha and test-retest, respectively. All questions were scored on a Likert Scales. Moreover, the unanswered questions or invalid answers were regarded as missing data and excluded from the data article.
Experimental Features	A score of zero means that the person does not do the activity under data article, whereas a score of 100 indicates that the person is highly engaged in doing the activity.
Location of Data Source	Kermanshah Province, Iran
Data Accessibility	Data were included in this article
Related research article	A.Ziapour,N.Kianipour, Health-related Quality of Life among University Students: The Role of Demographic Variables, J. Clin. Diagn. Res. 12(2017) JC01 - JC4[1].

**Value of the data**

• In the data of the present data article, the status of students’leisure time and health-related quality of life at Kermanshah university were investigated. Hence, the data of the present data article can be used by health authorities and decision-makers in the relevant area.

• The obtained data of the present data article demonstrated that there was a significant relationship between leisure time and health-related quality of life. It is suggested that university administrators and plannersimprove the health of students and improve their quality of life through holding workshops and training courses with the aim of enhancing the usefulness of students׳ leisure activities.
• The obtained data of this data article can be used to improve the leisure time, health-related quality of life and the interest of students in research at KUMS.

## 1 Data

Of the whole 420 subjects, 220 subjects (52.4%) were female and 200 subjects (47.6%) were male, and the average age of participants was 21.96 ± 3.70. In addition, the under-20 age range was in the majority (221 subjects or 52.6%). In terms of marital status, the majority of participants were single (390 subjects or 92.9%). Besides, in terms of education, 339 subjects (80.7%) were doing a bachelor׳s degree, and the majority of samples were majoring in nursing and midwifery (198 subjects or 47.1%). Moreover, in terms of the Residence, 201 subjects (47.9%) were living in dormitories, and the majority of students were born in urban areas (316 subjects or 75.2%) (Table 1).

**Table 1:** The participants׳ demographic characteristics.

**Variables**	**Groups**	**Number (%)**
Gender	Male	200 (47.6%)
	Female	220 (52.4%)
Marital status	Single	390 (92.9%)
	Married	30 (7.1%)
Place of birth	Urban Areas	316 (75.2%)
	Rural Areas	104 (24.8%)
Age (years)	20≥	221 (52.6%)
	20≤	199 (47.4%)
Residence	Dormitory	201 (47.9%)
	Rental	79 (18.8%)
	Personal	140 (33.3%)
Education	Associate׳s degree	42 (10%)
	Bachelor of Arts	339 (80.7%)
	Master of Arts	39 (9.3%)
Faculty	Paramedics	119 (28.3%)
	Nursing and Midwifery	198 (47.1%)
	Public Health	103 (24.5%)

The mean and standard deviation of the total score of students’ leisure time measured 3.25 ± 0.61. The obtained data also demonstrated that in-person communication and training classes had the highest and lowest relationships with the total quality of life (3.40 ± 0.72 and 3.14 ± 0.80, respectively).It was found that the mean and standard deviation of the total score of students’ quality of life measured 2.50 ± 0.41. As for the health-related quality of life of samples, the obtained data indicated that vitality and limitations related to physical problems had the highest and lowest means and standard deviations (3.53 ± 1.18 and 1.18 ± 0.19, respectively) (Table 2).

**Table 2:** The means and standard deviations of the total scores of students’ quality of life and leisure time and their respective components.

**Statistical Indexes**		**Mean ± SD**
**Indexes**	**Sub-indexes**	
Leisure time	In-Person communications	3.40 ± 0.72
	Studying	3.23 ± 0.74
	Artistic activities	3.31 ± 0.67
	Unplanned activities	3.18 ± 0.71
	Sports activities	3.17 ± 0.82
	Religious activities	3.27 ± 0.69
	Recreational activities	3.34 ± 0.84
	Training classes	3.14 ± 0.80
	Total leisure time	3.25 ± 0.61
Health-related quality of life	Physical functioning	2.83 ± 0.33
	Limitations related to physical problems	1.18 ± 0.19
	General health perceptions	2.31 ± 0.43
	Vitality	3.53 ± 1.16
	Mental health	2.87 ± 0.96
	Social role functioning	3.10 ± 1.28
	Bodily pain	2.49 ± 1.3
	Emotional role functioning	1.73 ± 0.26
	Total health-related quality of life	2.50 ± 0.41

The obtained data demonstrated that the relationship between the total leisure time and total quality of life of students was positive and significant. It was found that in-person communication had the highest relationship with the total quality of life. In contrast, no relationship was found between the artistic activities and the total quality of life (Table 3).

**Table 3:** The correlation between leisure time and health-related quality of life of university students.

**Hypothesis**	**Leisure time**	**Health-related quality of life**	**(r)**	**(N)**
1	Recreational activities	Total HRQOL	0.336**	420
2	Studying	Total HRQOL	0.229**	420
3	Artistic activities	Total HRQOL	0.059	420
4	Unplanned activities	Total HRQOL	0.212**	420
5	Sports activities	Total HRQOL	0.243**	420
6	Religious activities	Total HRQOL	0.163**	420
7	In-Person communications	Total HRQOL	0.349**	420
8	Training classes	Total HRQOL	0.137**	420
–	Total leisure time	Total HRQOL	0.206**	420

** Correlation is significant at the 0.01 level (2-tailed).

## 2 Experimental design, materials and methods

Here, 420 students at Kermanshah University of Medical Sciences were studied in 2017. Moreover, research samples were selected in two stages. In the first stage, out of seven colleges affiliated to Kermanshah University of Medical Sciences, three colleges (health, nursing and midwifery) were selected, and in the second stage, the samples were selected through multistage cluster sampling and Cochran sample size formula.

Further, the ethical principles employed included obtaining the necessary permits, retaining the rights for the schools being researched to either accept or reject to participate, and ensuring the confidentiality and non disclosure of the personal information of samples. Then questionnaires were distributed among the target sample. To this end, the objectives were explained to the target subjects Informed consent was obtained from all participants before data was collected. Not to mention, the exclusion criteria were the samples disinterested in participation in the study and handing over incomplete questionnaires.

As for data collection, three questionnaires were used: a demographic questionnaire, a researcher-made leisure time questionnaire, and the health-related quality of life questionnaire by Ware and Sherbourne.

Demographic Questionnaire: It consisted of seven items: gender, age, place of birth, marital status, residence, education, and faculty.

Leisure Time Questionnaire:This 45-item instrument was developed by the researcher to assess the status of spending leisure by the samples under data article. In the formulation of this questionnaire, the studies conducted by the National Youth Organizations about the young׳s leisure time and lifestyles were reviewed [2], [3], [4], [5], [6], [7], [8], [9], [1], [10], [11], [12], [13], [14]. The questions were carefully designed and tailored to the students’ conditions. The subscales of this questionnaire were in-person communications (3 items), training classes (6 items), recreational activities (14 items), data article (3 items), artistic activities (3 items), unplanned activities (5 items), sports activities (5 items), and religious activities (6 items). Furthermore, the questions were answered on a five-point Likert scale: everyday (100), several days a week (75), several days a month (50), rarely (25), never (0).Each question had a score in the range of zero to 100. A score of zero means that the person does not do the activity under data article, whereas a score of 100 indicates that the person is highly engaged in doing the activity.The face and content validities of the instrument were confirmed by the health and sociology experts. In addition, the reliability of the instrument was confirmed through calculating the Cronbach׳s alpha (0.84).

36-item Short Form Survey (SF-36):This 36-item scale, developed in America by Ware and Sherbourne [1], [15], [16], [17], [18] was designed for assessing health and quality of life.The subscales of this questionnaire were physical functioning (10 items), limitations related to physical problems (4 items), emotional role functioning (3 items), vitality (3 items), mental health (6 items), social role functioning (2 items), bodily pain (2 items), general health perceptions (6 items) [16], [19], [20]. A score ranging from 0 (indicating the worse health status) to 100 (indicating the best health status) was assigned for each domain [18]. Furthermore, the Cronbach׳s alpha was used to determine the reliability of this instrument (0.85) [21]. Additionally, this instrument was standardized by Montazeri et al. [22]. in Iran, too (0.70).

Furthermore, for data analysis, the descriptive (frequency distribution, mean, and standard deviation) and inferential statistics (Pearson correlation coefficient) were employed in the SPSS Statistics Software Version 23.0, and it should be noted that the related presumptions were examined prior to the above-mentioned tests.
